# Structure, mechanics and material properties of claw cuticle from mole cricket *Gryllotalpaorientalis*

**DOI:** 10.1371/journal.pone.0222116

**Published:** 2019-09-06

**Authors:** Zhifeng Zhang, Yan Zhang, Junxia Zhang, Yueying Zhu

**Affiliations:** Tianjin Key Laboratory of Integrated Design and On-line Monitoring for Light Industry & Food Machinery and Equipment, College of Mechanical Engineering, Tianjin University of Science & Technology, Tianjin, China; University of Western Ontario, CANADA

## Abstract

Powerful shovel-like forelimbs with special shape, structure and biological materials enable mole cricket to digging efficiently. During digging, the tip of the claw needs to wedge into the soil, and the base needs to withstand considerable anti-shear force. In this study, we analysed the structural characteristics, material composition and mechanical properties of the claw teeth using scanning electron microscopy, plasma atomic emission spectroscopy, nanoindentation and finite element analysis. The results show that the tips of claw teeth have a dense and homogeneous structure and a higher hardness and contents of Mn and Zn compared with the base. The structure of the base of claw teeth has an obvious laminar structure and higher fracture resistance. Moreover, it is speculated from the simulation results that basal position of the claw teeth is tough enough to withstand high stress, and the presence of the ribs effectively improves the mechanical stability and load-bearing capacity of the teeth during excavation. The results of this study can provide inspiration for the design of efficient mechanical components and agricultural implements.

## Introduction

Mole crickets are typical soil insects that possess an excellent excavating ability. The digging speed of mole crickets can reach 20 cm/min in soft soil, which equals 5 times their body length (approximately 4 cm)[1]. The efficient excavation capacity of mole crickets is related to the geometry, structure and material of the claws on the forelimbs that is used as the main soil-engaging component[2,3]. The shape of the claw is similar to a digging shovel and has a concave upper surface and has ridge-like protrusion at the lower surface. Each claw has four claw teeth that are arranged in parallel on the tip of the claw, and there is a 30° angle between each tooth. The tip blade of a claw tooth is rounded and has a strong ability to wedge into soil[4].

**Fig 1:**
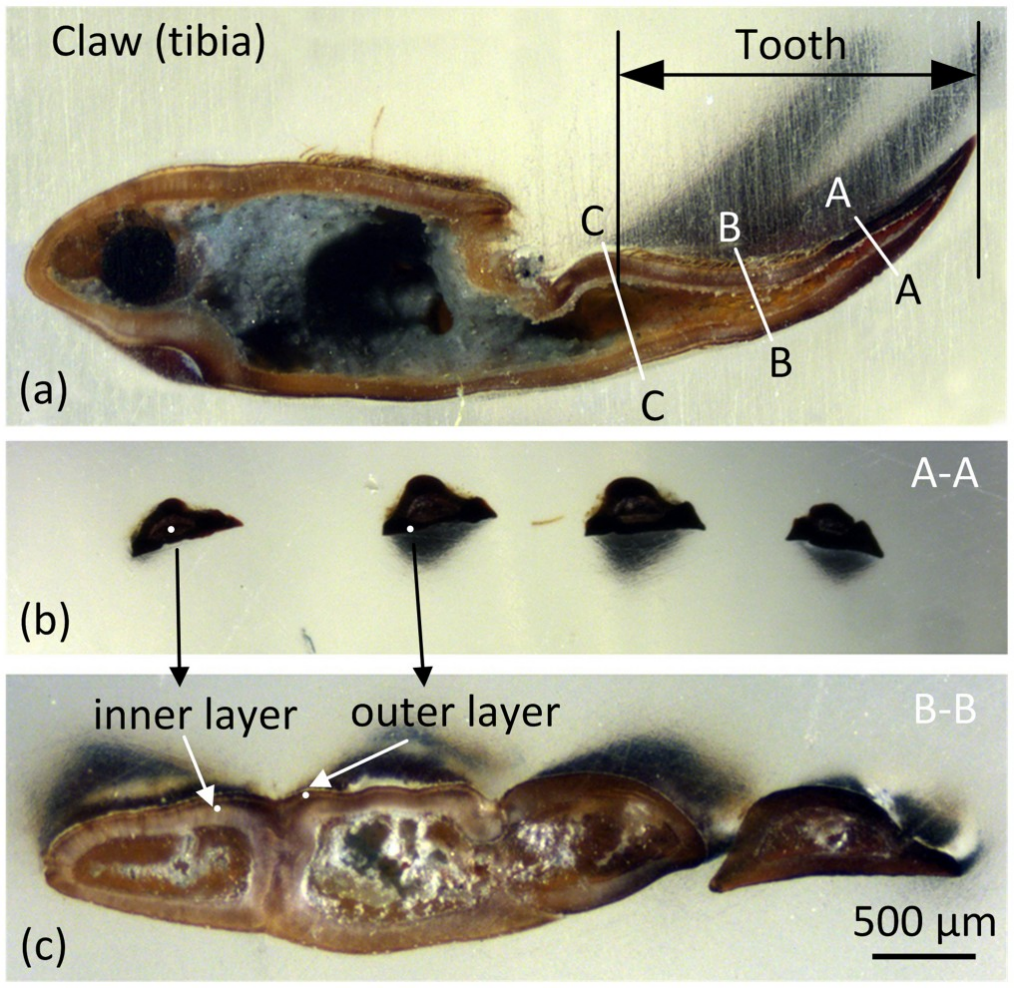
Stereoscopic images of the section of claw tooth. (a) The longitudinal section of 3rd claw tooth of forelimb of mole crickets, (b) a cross section at the 1/3 tooth length (from tip to base), (c) a cross section at the 2/3 tooth length (from tip to base).

The claws must withstand considerable resistance and the applied digging force toward soil. Therefore, the shovel-like forelimb is exceptionally large compared to common insects. The external shape of a claw tooth is wedge-like, and the cross-sectional shape is approximately triangular. Fig 1a shows that there is a cavity in the claw tooth. The cross-section of the cavity area gradually increases from tip to base along the longitudinal direction[5]. The tips of the claw teeth are dark brown or black, and they have a smooth surface and no hairs. Conversely, the base colour of the claws is light brown and is covered in dense hairs. This difference may be related to its structure, mechanics, and material properties. For example, there is a similar phenomenon observed in the front claws of stone crabs, and the tip colour is significantly darker than the base. Melnick et al.[6] conducted a series of mechanical tests and showed that the deep region of the claw was harder and tougher than the light region. Additionally, scanning electron micrograph tests showed that the microstructure of the deep region had a lower porosity. Similarly, the tip colour on the jaw teeth of sea worms is much darker than that of the base. Lichtenegger et al.[7] found that the tip areas had higher zinc content and higher local hardness and stiffness and that the zinc-containing compounds in the jaw teeth were involved in the formation of microstructures.

In summary, it can be hypothesized that there are differences in mechanical properties between the tip and base of the mole cricket’s claw teeth due to the differences of the interaction with soil and the colour appearance. In this study, we analyzed the structure, material and mechanical properties of the tip and base of the mole cricket’s claw teeth using scanning electron microscopy, nanomechanical tests, plasma atomic emission spectroscopy and finite element analysis. We then describe the relationships among the function, exterior colour[8], mechanics and structure of the claw teeth. Moreover, we employed a realistic claw model with material properties to analyze the relation between biomechanics and digging behavior using finite element simulation[9]. This research can provide a theoretical basis for designing modifications to the shape and properties of bionic soil-engaging components.

## Materials and methods

### Terminology

**Fig 2:**
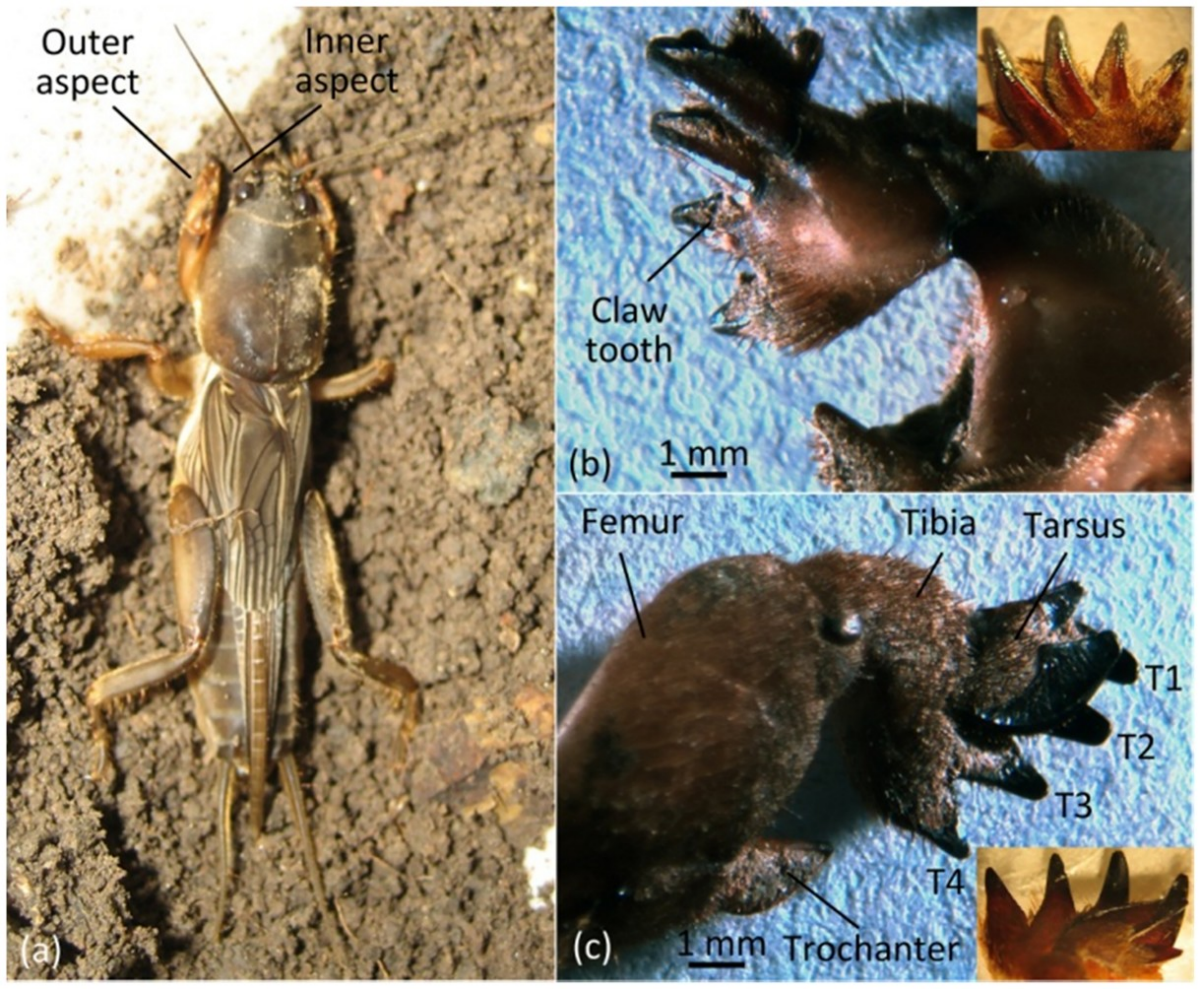
Forelimb of mole cricket. (a) Mole cricket, (b) inner aspect of the right claw, (c) outer aspect of the right claw.

The forelimbs of mole crickets have evolved into a digging leg that consists of femur, trochanter, tibia, tarsus, etc (Fig 2). The tibia of the forelimb is a specialized digging tool and contains 4 teeth (T1 to T4). The inner aspect of claw teeth is the back of the soil-contacting surface, which is rough with a rib in the middle through the tip to basal (Fig 2a). On the longitudinal profile, the inner aspect is the side of the outer arc and is the side of symmetrical convex arc on the transverse profile (Fig 1a and 1c). The outer aspect of a claw is the outward side away from the body and is the soil-engaging surface. The longitudinal profile shows that the outer aspect of the side has a concave arc and that the side makes a nearly straight line on the transverse profile (Fig 1a and 1c).

### Scanning electron microscopy

Mole cricket samples were collected in Daodi Town (39°32′N, 118°11′E), Tangshan City, Hebei Province, China. No specific permits were required for the described field studies, as the location where samples were collected is not privately owned or protected. The field studies did not involve endangered or protected species. Two 1st claw tooth samples were removed and collected. The samples were washed in distilled water by using an ultrasonic cleaning machine to remove impurities and were then dehydrated and dried with medical absorbent cotton and silica gel desiccant. After frozen in refrigerator with the temperature of -18°C for 24 h, two tooth samples were broken off manually at the 1/3 (Fig 1a (A-A)) and 2/3 (Fig 1a (B-B)) of tooth length (from tip to base). The fractured samples were attached to the sample stage with the fracture section face up and the fragments coated with gold was carried out by an ion sputtering apparatus (JSC-1100, JEOL, Japan), then observed by scanning electron microscopy (JSM-5310, JEOL, Japan).

### Nanoindentation test

The mechanical properties of the claw teeth of mole crickets were determined by nanoindentation test, which is an efficient method to evaluate the mechanical properties of materials. The nanohardness and reduced modulus can be determined according to the load-displacement curve[10]. In order to avoid the influence of the recovery process of the elastic deformation of the indenter after lifting on the actual elastic modulus of the specimen, all the elastic modulus values of the specimens were derived from the reduced modulus based on the Oliver-Pharr method[11]. The relationship between reduced modulus, elastic modulus and Poisson’s ratio of sample material and indenter material is as follows,

$$\frac{1}{E_{r}}\;=\;{\left[\frac{1\;-\;v^{2}}{E}\;\right]}_{specimen}\;+\;{\left[\frac{1\;-\;v^{2}}{E}\;\right]}_{indenter}$$ (1)

in which, *E*_*r*_ is reduced modulus, *E* is elastic modulus, and *ν* is Poisson’s ratio.

Before the nanoindentation test, the claw tooth samples were prepared, cleaned and dried by natural dehydration. After measuring the length, the samples were embedded in epoxy resin and fixed for 48 h in order to be mechanically stabilized.

The raw cross-sections at two positions located at 1/3 (Fig 1a (A-A) and 1b) and 2/3 (Fig 1a (B-B), and 1c) of the length were obtained by abrasive grinding normal to the intended measuring plane. Then, the cross-sections were polished using a cross-section polisher. The nanomechanical properties of the different parts of the tip and base section were measured using the nanoindentation test system (Agilent, nanoindenter G200, USA) with a Berkovich diamond tip. The trapezoidal loading method was used with the packing time of 10s. The strain rate was 0.05 nm/s, and the maximum indentation depth was 2000 nm.

In order to study the mechanical properties of different parts of the claw teeth, 16 claw samples were collected and divided into two groups for the nanomechanical test. In one group, the mechanical properties of four positions on the cross section were tested, including exocuticle and endocuticle positions respectively at the tip and base of claw teeth[12,13]. In the other group, the mechanical properties along the longitudinal direction of the claw teeth were measured, and the three positions were located between the tip and cross-section A, the position between cross-sections A and B, and the position between the cross-sections B and C, respectively (Fig 1a). Three points for each position were measured. All the data were statistically analyzed by SPSS 18.0 software, two-way ANOVA was used for the comparison analysis between groups, and Bonferroni correction was used to adjust for multiple comparisons. Quantitative data was expressed as mean**±**standard deviation (mean±SD).

### Element content test

Ten samples of the T1 claw tooth were collected from different individuals. The tip parts of the claw teeth were separated from the base parts and were then cleaned by an ultrasonic cleaner separately for 1 min before drying at 100 °C to a constant mass. The samples were then ground to powder using an agate mortar and were completely boiled with HNO_3_ + HClO_4_ concentrated acid (volume ratio 87:13)[14]. The tests were conducted by an inductively coupled plasma atomic emission spectroscopy (Optima 3300 DV, Perkin-Elmer, USA). The elements Ca, Mg, P, Fe, Mn and Zn in the tip and base parts of claw teeth were measured.

### Finite element analysis

**Fig 3:**
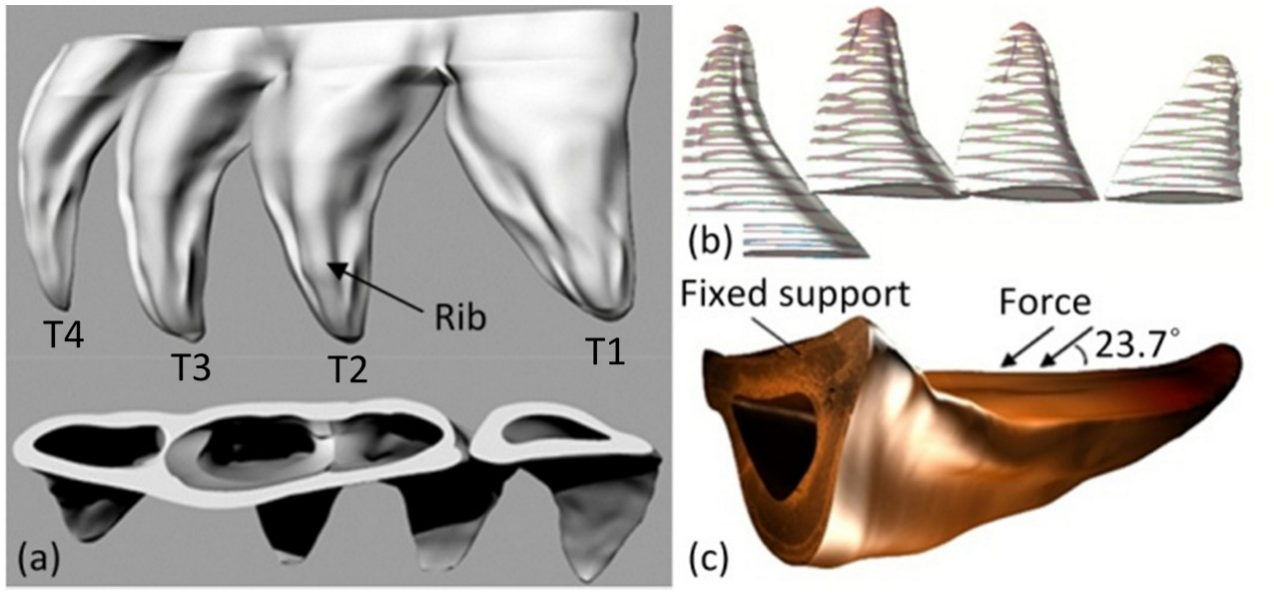
The reconstructed three-dimensional model and the initial conditions. (a) The reconstructed model of claw teeth; (b) the cross-section slices; (c) the initial conditions.

**Fig 4:**
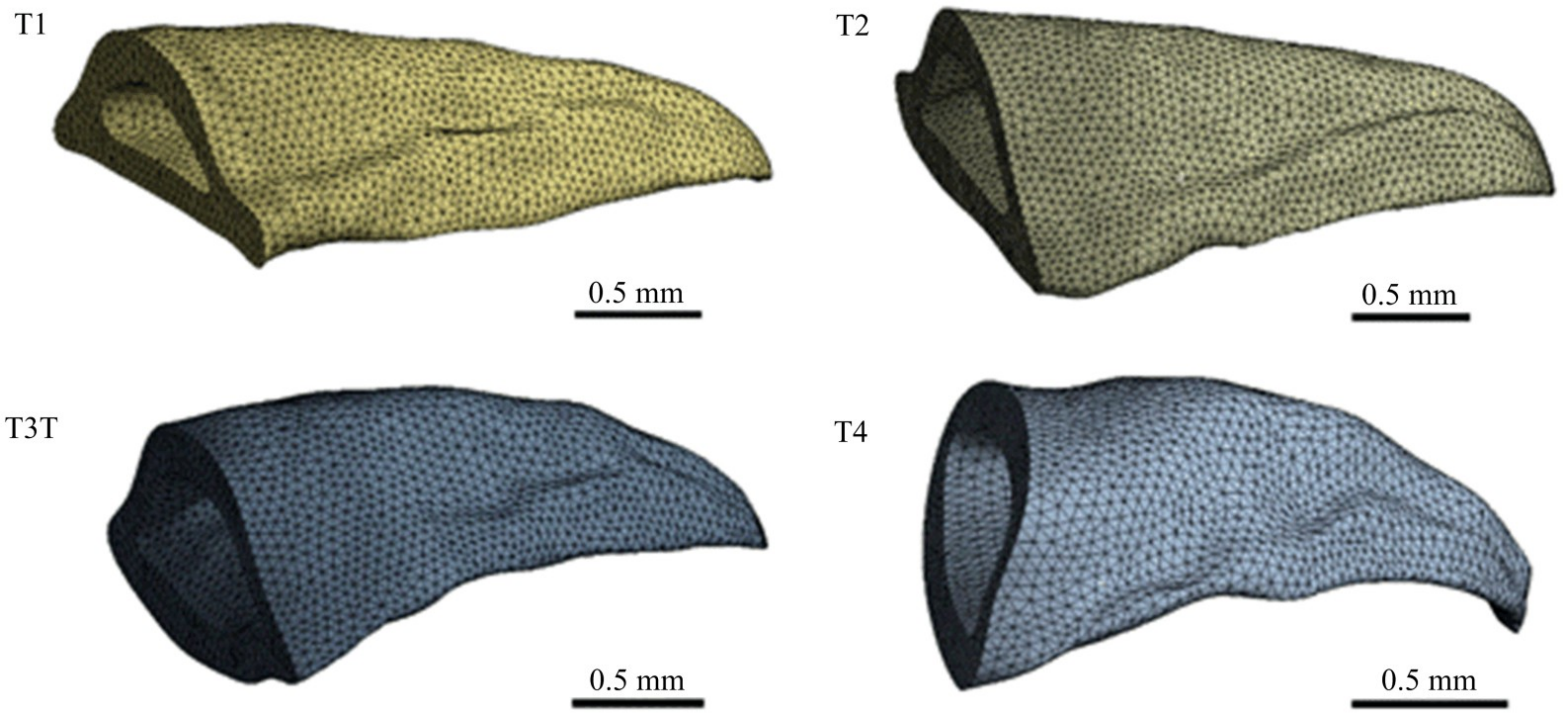
The meshed claw tooth models by quadrilateral meshes.

Three-dimensional models of T1 to T4 claw teeth were respectively established by reverse engineering method based on the inner and outer contour curves obtained from 17 cross-section slices. The method for mechanically obtaining the cross-sections has been described in the earlier section. During this process, the grinded thickness was measured by a micrometer to obtain the layer distance between two cross-sections (Fig 3b), and the image of every cross-section was captured by a digital stereomicroscope. The 3D models of claw teeth were constructed based on the captured cross-section images using Rhinoceros software. The static loading was used to analyze the stress condition of the tooth model during excavation. Our previous study has shown that the maximum angle between the two forelimbs was about 47.4 degrees[15]. At this condition, the forelimbs are fully extended, the claws, the tibia and the femur are in a straight line, and the angle between the force acting on the tooth and the tooth surface is about 23.7 degrees. In addition, the volume of the T2 claw tooth is about 2.837 mm^3^ (Fig 3a), and the mass measured by an electric balance is 3.50±1.04mg, so the average density of the claw teeth is about 1.23×10^−3^ g/mm^3^. In the finite element analysis, the model was meshed by quadrilateral meshes with an element size of 5.0×10^−2^ mm (Fig 4). The mesh quality metrics was checked, and the average mesh qualities of T1, T2, T3 and T4 were 0.82795, 0.82815, 0.82763 and 0.82263, respectively. These values are higher than the empirical value (0.7), indicating that the mesh quality is acceptable. The fixed pattern of the model was bottom fixed support (Fig 3c), and the stiffness behaviour of geometry was defined as nonlinear flexible solid. The Poisson’s ratio of insect epidermal biomaterials is typically 0.25[16]. The elastic modulus value of each tooth was obtained from the nanoindentation results. The uniform load is the reaction force of the claw tooth during excavating, which was applied on the group of elements to the lateral surface of claw teeth (Fig 3c). The value of the reaction force is about 0.84 N that was obtained in our previous study[15].

## Results and discussion

### Structural characteristics of claw tooth exoskeleton

**Fig 5:**
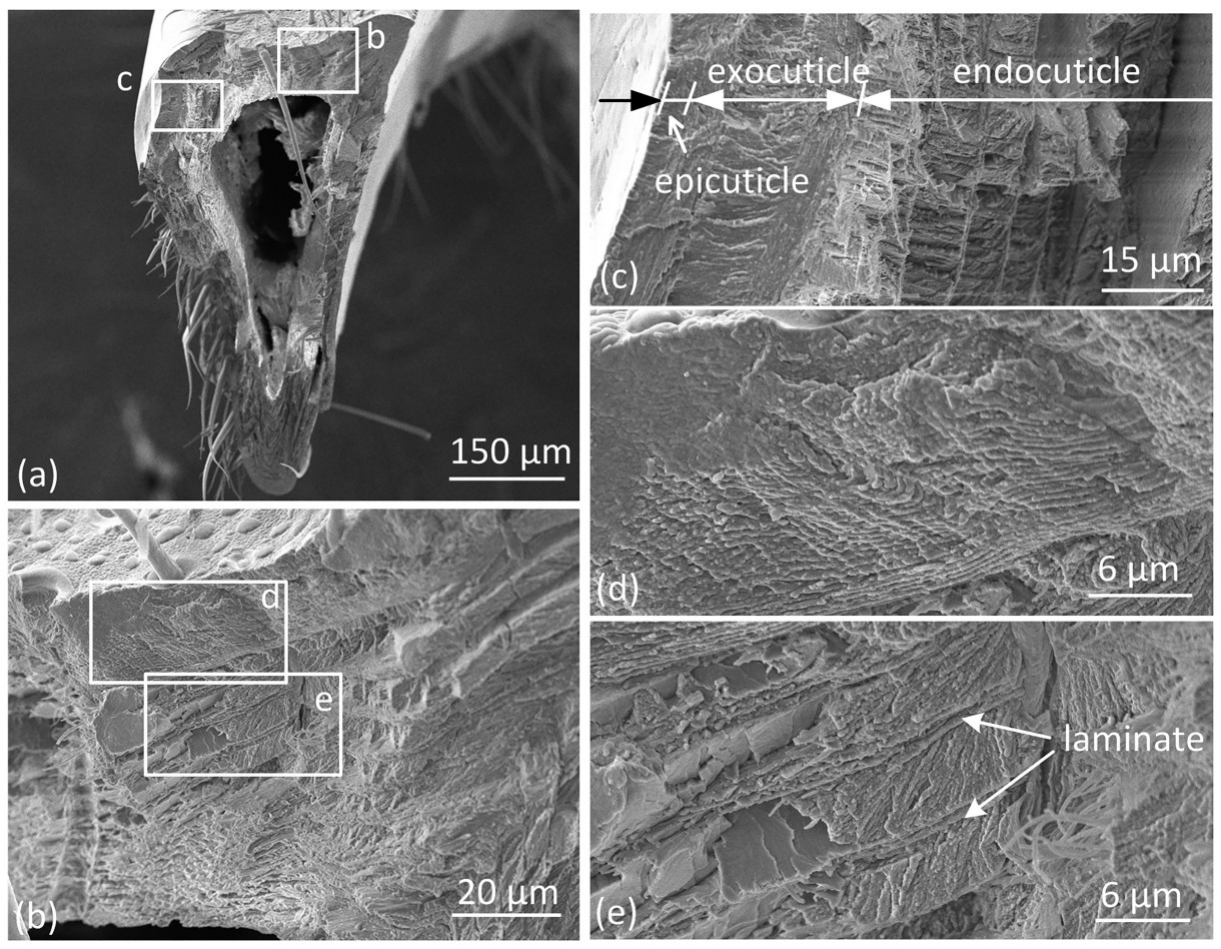
SEM images of the fracture cross section of base region of claw teeth.

**Fig 6:**
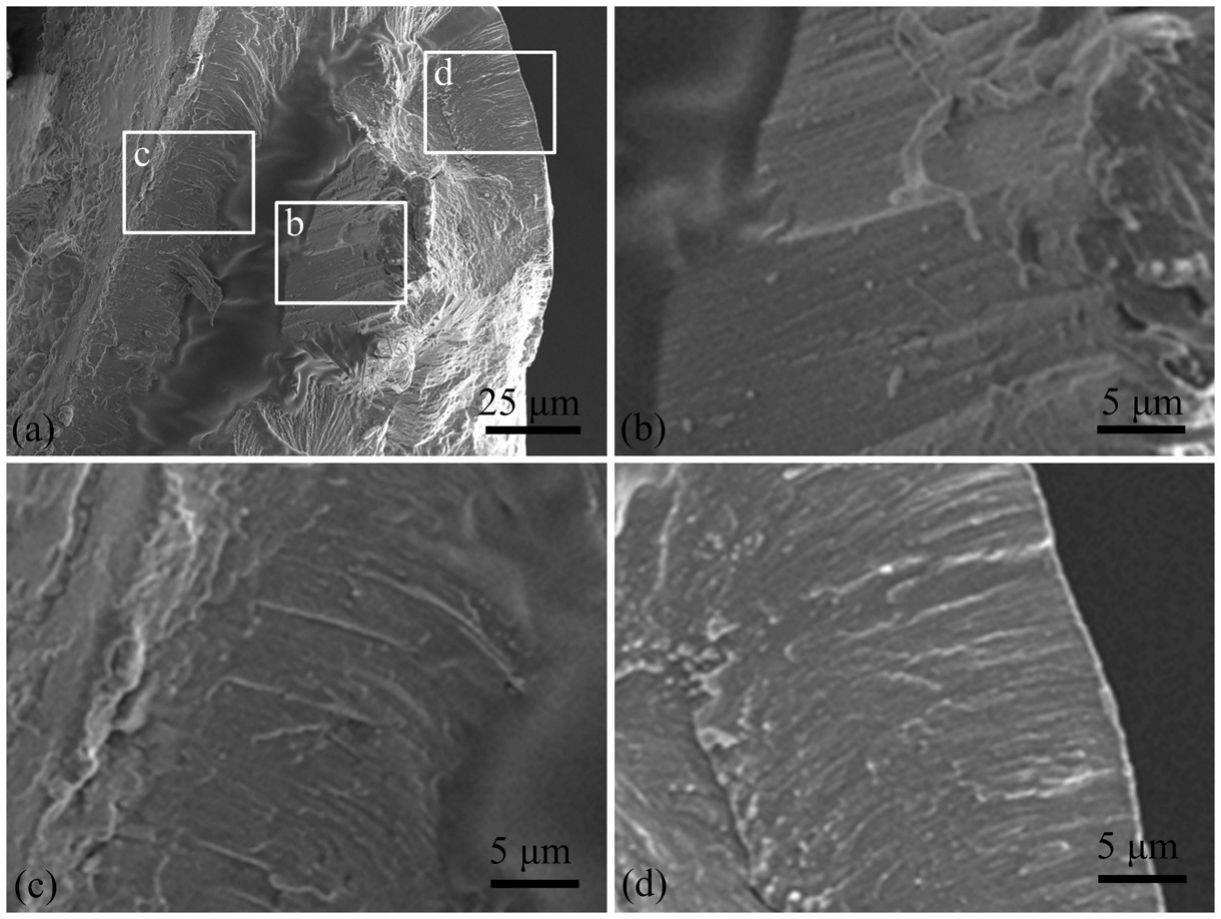
SEM images of the fracture cross section of tip region of claw teeth.

The claw tooth of mole crickets has a sharp tip and a thick base, which forms a wedge shape. The cross-sectional shape of claw teeth is approximately triangular. There is a triangular cavity inside the claw tooth that forms a shell-like structure (Figs 5 and 6). The hollow structure has obvious advantages over solid structure in bearing loads when the total amount of the material is constant; moreover, the triangular cross-section makes the structure more bending resistant than it would have been with other cross-section (e.g. circular) [17,18].The area ratio of the cavity on the cross section increases with the distance from the tip to end[5]. Additionally, the inner aspect exoskeleton of teeth is thicker than the outer aspect, especially at the ribs and corner positions. The exoskeleton of insects is composed of a composite material with a gradient multilayer structure[19,20]. The outermost layer of the exoskeleton is termed epicuticle, which is a thin wax layer that provides the main waterproof barrier to the environment. The procuticle is located under the epidermis and is the main structural part of the exoskeleton that is used to withstand the mechanical load from the environment. The procuticle is further divided into the exocuticle and endocuticle, which are similar in structure and material composition. However, the texture of the exocuticle is stacked more densely, as shown in Fig 5c.

Fig 5 shows the fracture cross-section of the base region of claw teeth. The procuticle ridge on the inner aspect is clearly visible. However, the boundary between the epicuticle and the procuticle is not obvious, as shown in Fig 5c. The structure of the exocuticle is uniform and dense, and the layers are densely stacked (Fig 5d). The structure of the endocuticle is less dense than the exocuticle. The upper part of the endocuticle shows the obvious corrugated board-like layered structure, and the layers are connected by laminates (Fig 5e). In the lower part of endocuticle, the structure gradually becomes looser with depth, and the porous fibres are assembled in a spiral stacked pattern (Bouligand structure). In the example shown in Fig 5c, the thickness of the exocuticle is approximately 15.4 μm, and the thickness of the endocuticle is approximately 45 μm; the ratio of the two layers is thus approximately 1:2.9.

The layered structure, transition of fibre orientation and irregular fracture surface of the cuticle of the claw tooth present as a laminate structure. The insect exoskeleton is a complex structure consisting of chitin fibres and protein matrix. The mechanical properties of the cuticle are determined by the properties of the protein matrix and the orientation of the chitosan fibres. The protein matrix can exhibit a great hardness range with a certain degree of hydration, and chitin fibres have different stacking patterns on the sub-micron scale, including parallel and helical patterns[21,22]. The Bouligand (helical stacking) arrangement provides structural strength and is in-plane isotropic[19].

Fig 6 shows the fracture cross section of the tip of claw teeth. The internal cavity is smaller than the base. The cuticle structure of the tip is not obvious, and there are some oriented structural differences between inside and outside of the cross section (Fig 6a). The stacking direction/growth direction of the material seems to be vertical to the outer surface in the outside of the cross section (Fig 6b), and parallel to the longitudinal direction of claw in the inside (Fig 6c).

The cuticle structure of claw teeth in mole crickets is a special form of exoskeleton and is similar to the cuticle of other body parts because the chitosan fibres are stacked in parallel or spiral models. The main function of the claw is digging. It is assumed that there are different structural forms in different locations, and the combination of structures is conducive to the realization of biological functions. The mole cricket uses a "digging-expanding" type of excavation[23], where the tip of the claw teeth is mainly subjected to compression and friction when wedging into soil and the base of the claw teeth is subjected to shear force in expanding motion. Upon comparing Figs 5 and 6, the chitosan fibres in the tip of claw teeth are arranged in a dense and parallel manner, which may helpful in with standing compressive stress. The cross-section in the base shows a distinct laminated structure with deflected and complex fibres, which may helpful in resisting fracture[24].

### Mechanical properties of claw teeth

#### The mechanical properties in cross section

**Fig 7:**
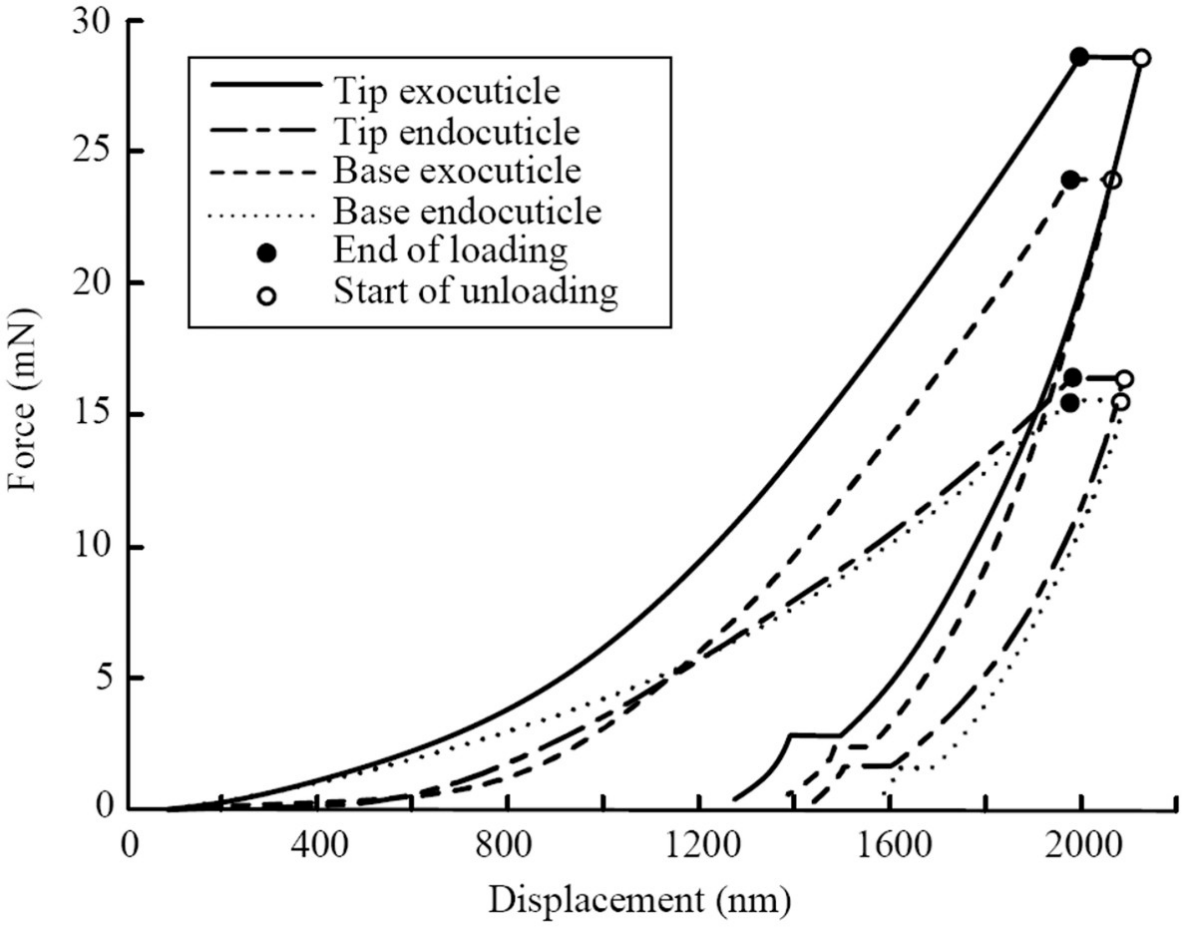
The load-indentation curve of nanometer mechanical properties.

**Fig 8:**
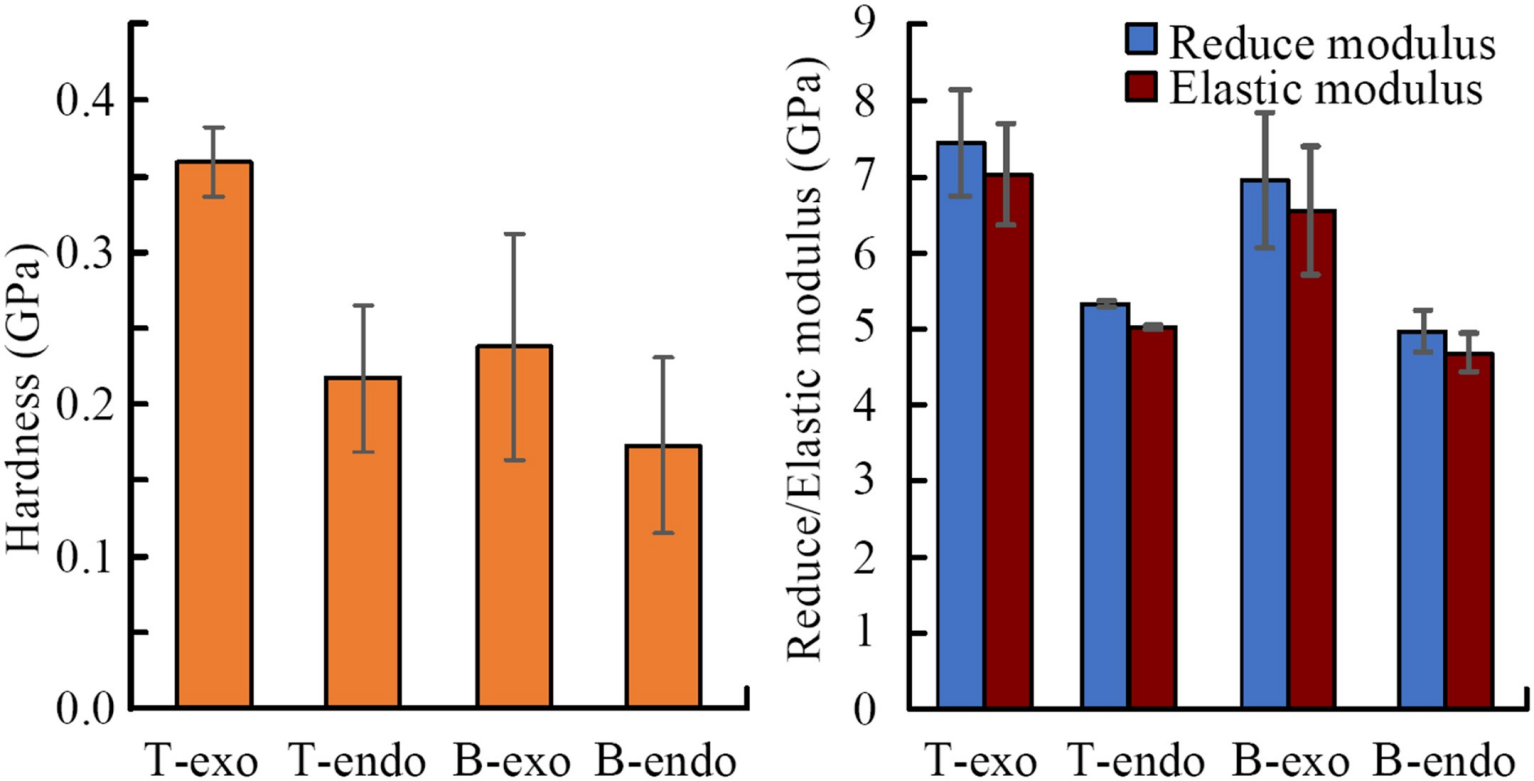
Mechanical properties comparison between the tip and base position. T-exo: tip-exocuticle (n = 4), T-endo: tip-endocuticle (n = 2), B-exo: base-exocuticle (n = 6), B-endo: base-endocuticle (n = 3).

Fig 7 shows the comparative of four single representative measurements. There is a clear difference in the structures of the exocuticle and endocuticle (Fig 5). Therefore, we conducted nanoindentation tests on four positions, including the exocuticle and endocuticle positions at the tip and base of the claw teeth. The results of the mechanical parameters are shown in Fig 8, and the load-indentation depth curves are shown in Fig 7.

As shown in Fig 8, the elastic modulus of the material of claw teeth is in the range of 4.7~7.0GPa, which is similar to 5~8.5 GPa of dried insect cuticle[25–27]. The comparison of the mechanical properties between the exocuticle and endocuticle in different part of claw teeth, as well as the comparison between tip and base part in different position of cuticle were listed in Table 1.

**Table 1:** The comparison of the mechanical properties in different locations of claw teeth.

Parameter	Comparison	Location	Results	P value
elastic modulus	exo vs endo	Tip	tip-exo>tip-endo	0.005*
		Base	base-exo>base-endo	0.002*
	tip vs base	Exo	tip-exo> base-exo	0.296
		Endo	tip-endo>base-endo	0.591
Hardness	exo vs endo	Tip	tip-exo>tip-endo	0.018*
		Base	base-exo>base-endo	0.145
	tip vs base	Exo	tip-exo> base-exo	0.009*
		Endo	tip-endo>base-endo	0.431

Note: exo denotes exocutile, endo denotes endocuticle, and the * denotes the statistic difference

The results show that the exocuticle of claw teeth has higher elastic modulus and nanohardness than the endocuticle, except the hardness in the base part. In the load-displacement curve (Fig 7), the slope of the unloading curve of tip-exocuticle is larger than that of tip-endocuticle. Therefore, for the measurements of these specific specimens, the tip exocuticle seems to have higher contact stiffness.

In addition, the exocuticle in the tip of claw teeth has a higher nanohardness than the base (P = 0.009), which indicating a higher material intensity. However, the differences of elastic modulus between tip and base are not statistically significant (all P>0.05), and the difference of endocuticle nanohardness between base and tip is also not statistically significant (P>0.05).

The higher hardness of the exocuticle enables the outer surface of the tooth tip to resistant abrasion and against stress under actual working conditions[28], and the low elastic modulus or stiffness of the endocuticle acts as a buffer to absorb stress and transmit forces, which is useful to endure the impact force from soil and protect the teeth from damage. A hardness tip may help claw teeth prevent compressing and wearing, and a low stiffness base may help protect claw teeth against fracture[29,30].

#### The mechanical properties of the four teeth

**Fig 9:**
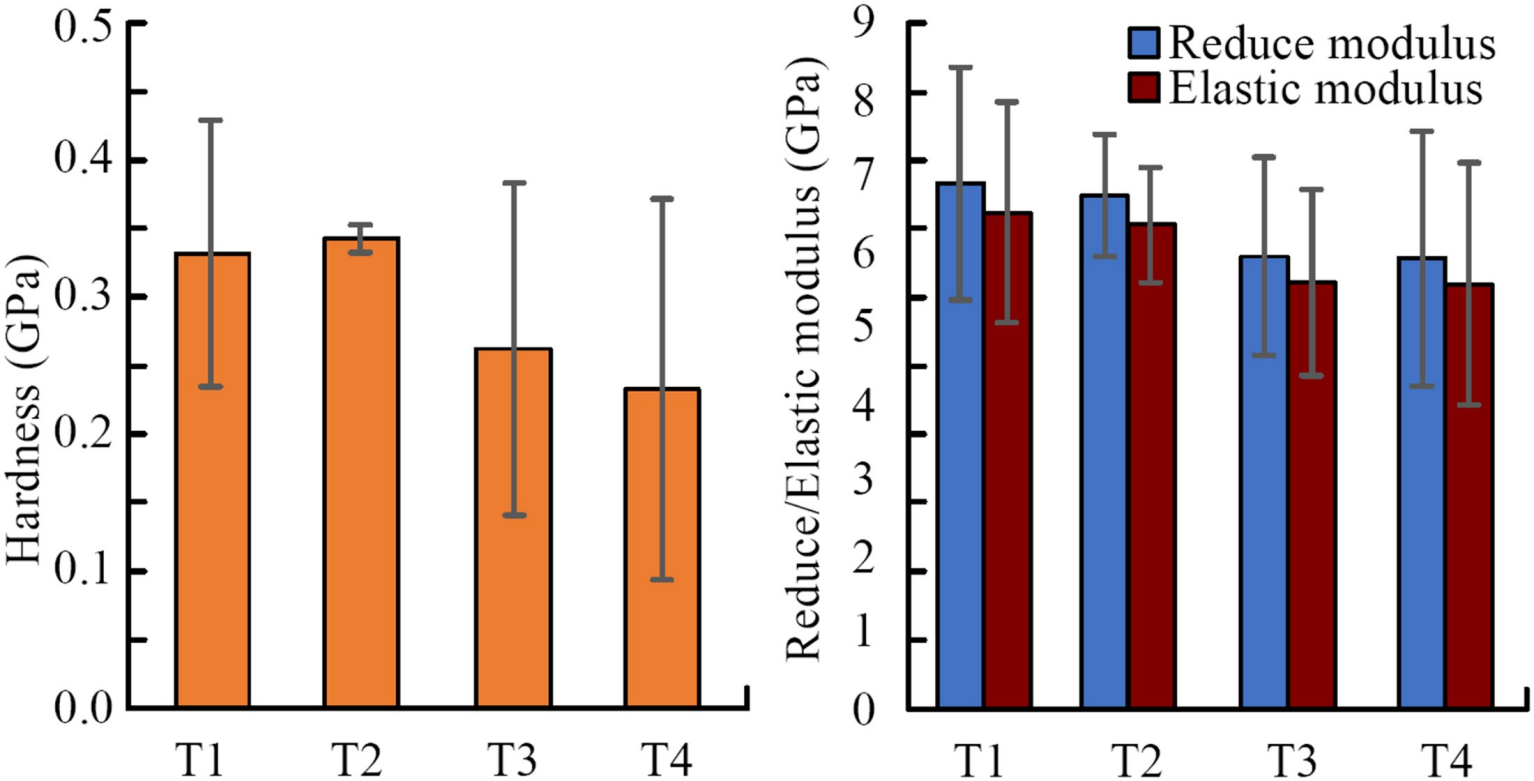
Mechanical properties comparison among the four claw teeth. T1: 1st claw tooth (n = 7), T2: 2nd claw tooth (n = 3), T3: 3rd claw tooth (n = 5), T4: 4th claw tooth (n = 3).

The mechanical properties of the four teeth were compared, which were measured by nanoindentor on cross-section of the teeth, as shown in Fig 9. The results show that the claw T1 and T2 has larger elastic modulus and hardness than T3 and T4, which may be related to the effect and contribution of each tooth in digging and excavating. However, the differences of elastic modulus between claw teeth is not statistically significant P = 0.5968 (P>0.05). This speculation can be explored in the future research work, but was not elaborated deeply in this study.

### Material composition analysis

Insects are covered by a rigid exoskeleton that supports the body, resists the external mechanical load, and prevents water loss. The insect exoskeleton is mainly composed of chitin, polysaccharide, structural protein, and inorganic minerals. In this study, the metal elements in the tip and base of claw teeth were analysed for Ca, Mg, Fe, Mn and Zn contents.

**Fig 10:**
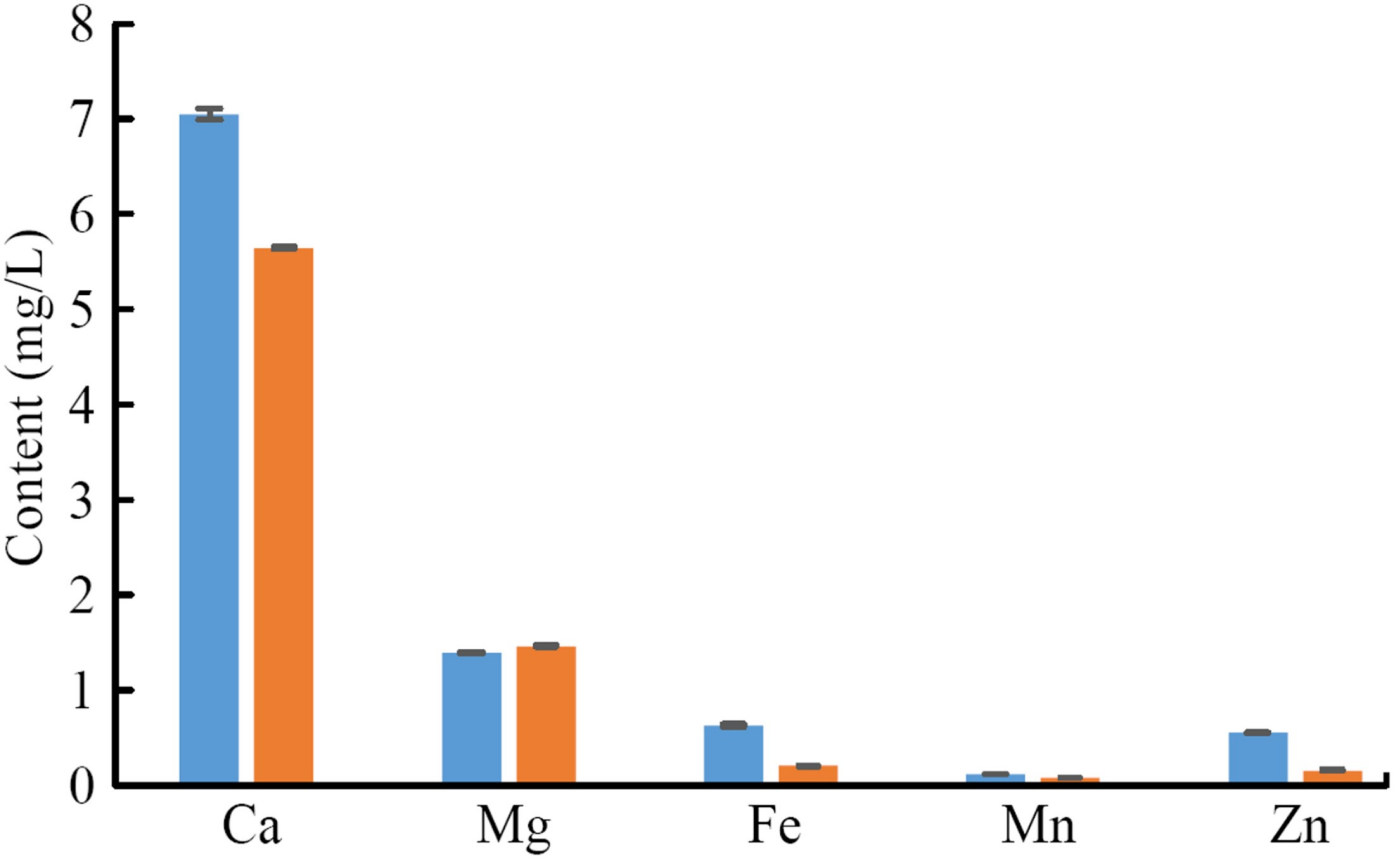
The content of metallic elements in claw teeth.

As shown in Fig 10, the material in the tip has a higher content in Ca, Fe, Mn, and Zn compared to the base (P<0.05). The content of Mg in the tip is slightly lower than that in the base (P>0.05). However, the metal element content in the claw tooth tip of mole crickets is higher overall, which is corresponds to the colour distribution.

Presence of metal ions[31], have been introduced as a strategy to increase the rigidity of organic composites. The available literature data indicate that the contents of Zn and Mn in the upper jaw of coleopteran insects are high and that there is a relationship between metallic element contents and the hardness of biomaterials[32]. The metal elements can increase the secondary bond between the materials, which helps form complexes with proteins and phenols. As a result, the density and fracture toughness of materials are enhanced. In addition, Morgan et al.[33] reported the hardness enhancing function of Zn and Mn in biomaterials. They speculated the metal elements played a role in enhancing localized hardness, abrasion resistance and fracture strength during evolutionary process[34,35]. Moreover, chemical gradients and cross-linking have been reported as another solution to enhance the mechanical properties of the organic models such as squid beak[18]. These gradients may cause variations in the optical properties of the tissues, as the dark colour in the tip of the claw. It is also reported that melanins can act as both oxidants and reductants, showing a high activity in binding metal ions[36].

### Finite element analysis results

**Fig 11:**
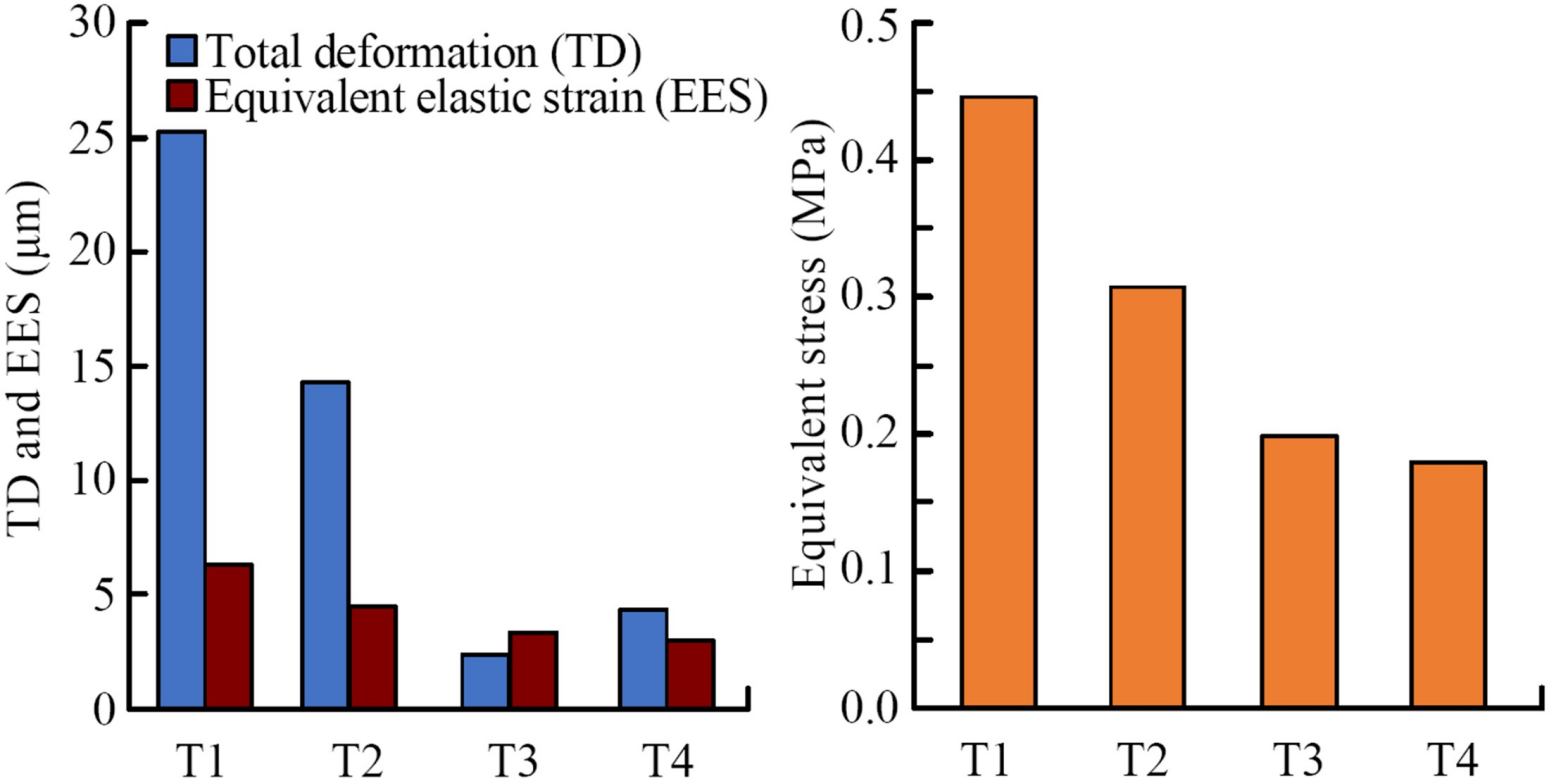
The maximum total deformation (TD), equivalent stress (ES) and equivalent elastic strain (EES) of four teeth by finite element analysis. T1: 1st claw tooth (n = 7), T2: 2nd claw tooth (n = 3), T3: 3rd claw tooth (n = 5), T4: 4th claw tooth (n = 3).

Fig 11 show the results of the finite element analysis, including the total deformation (TD), the equivalent stress (ES) and the equivalent elastic strain (EES) of the four claw teeth. The TD diagrams in Fig 7 show the maximum shape variables of the four claw teeth, the grey part indicates the original state of the teeth. The results show that the stress conditions of the four teeth are different in the excavation process, and the maximum value of ES, EES and TD of the claw T1 is the largest of the four teeth.

**Fig 12:**
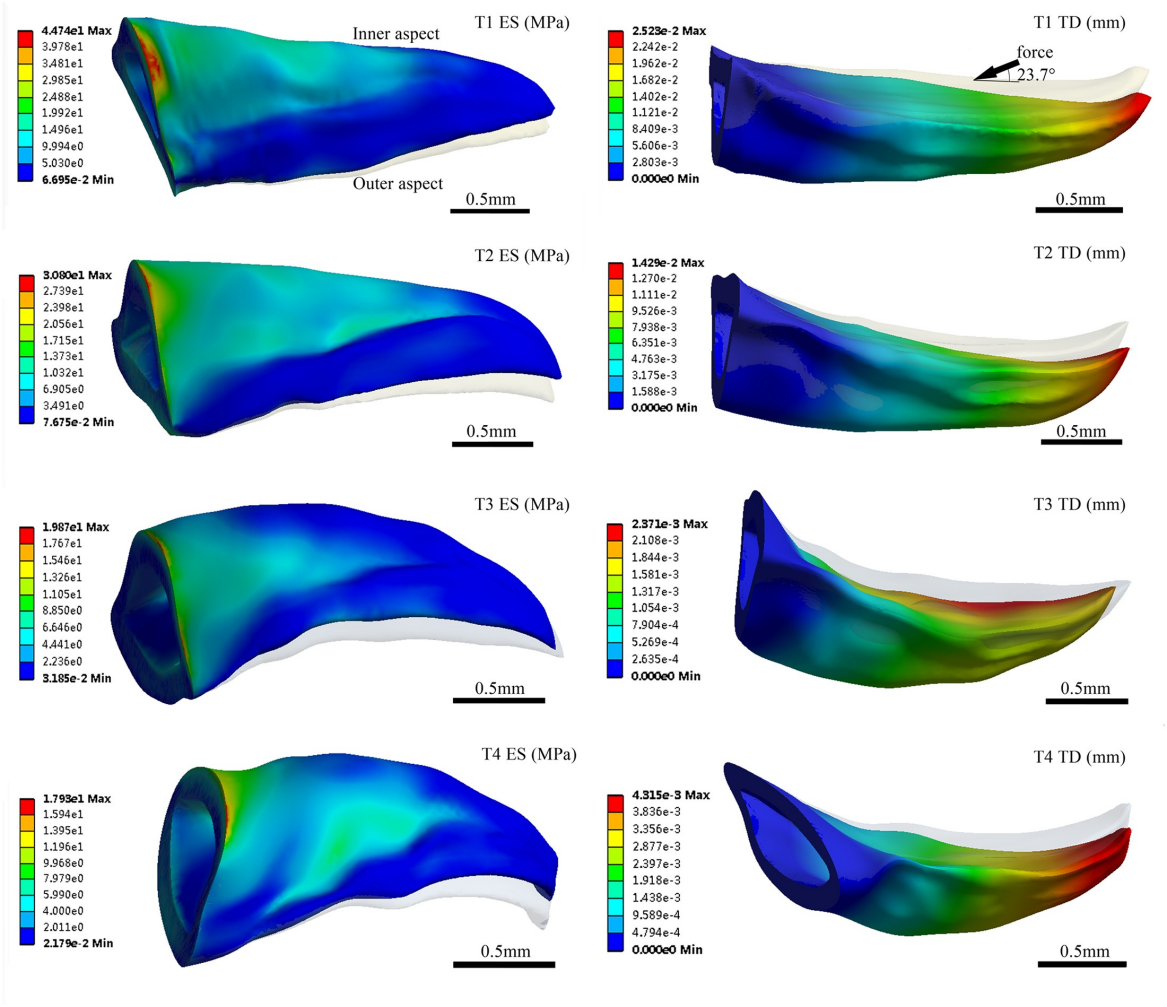
Simulation results of total deformation and equivalent stress.

The four claw teeth have the similar mechanical and deformation features in excavation, i.e. there is a large ES near the basal position and a large TD at the tooth tip for all the teeth(Fig 12). In excavating process, the material and the structure of the basal position of the claw teeth is speculated to be tougher than the tip position in order to withstand greater stress. Besides, the stress distribution on the outer side of the claw teeth is small, and the maximum stress appears on the inner upper edge of the basal position.

The inner side of the claw teeth is subjected to a larger stress, and the stress value gradually decreases from the rib to the edge, which indicates that the presence of the ribs effectively improves the mechanical stability and load-bearing capacity of the teeth during excavation. In fact, ribs are the most common reinforcing structures in living organisms[35].

## Conclusions

In this study, we evaluated the structural characteristics, material composition, and mechanical properties of different positions of the claw tooth from mole crickets using scanning electron microscopy, plasma atomic emission spectroscopy, nanoindentation test and finite element analysis. The relationships among these aspects were also described. The results show that mole cricket claws have evolved structural and material properties for soil digging. The structure of the tip part of claw teeth is densely uniform, their hardness value is higher, and its Mn and Zn contents are higher than those of the base. In the base part of claw teeth, the structure shows a more laminar stacking and better fracture resistance characteristic. The stacking density of the lamellar structure in the procuticle of claw teeth is significantly related to its mechanical properties, which shows an increase in hardness and elastic modulus of the material from the endocuticle layer to the exocuticle layer. This result is related to the excavation function of the claw of mole cricket. Besides, it is speculated from the simulation results that basal position of the claw teeth is tough enough to withstand high stress, and the presence of the ribs effectively improves the mechanical stability and load-bearing capacity of the teeth during excavation. However, in the simulation we simplified the claw teeth into a uniform material, which is inconsistent with the actual gradient structure. In further study we will improve the tooth model so as to improve the accuracy of the simulation. The results of this study can provide inspiration for the design of efficient mechanical components and agricultural implements.

## Supporting information

S1 Table The experimental results of nanoindentation as shown in Figs 8 and 9. (XLSX)

S2 Table Static analysis results of four claw teeth as shown in Figs 11 and 12. (ZIP)

S1 File Model of claw teeth as shown in Figs 3, 4 and 12. (RAR)
