# Supplementary material for: Structure, mechanics and material properties of claw cuticle from mole cricket Gryllotalpaorientalis
Source: PLoS One. 2019 Sep 6;14(9):e0222116. doi: 10.1371/journal.pone.0222116 (PMC6730930; doi:10.1371/journal.pone.0222116)
Supplement: S2 Table — (ZIP) [file pone.0222116.s002.ZIP › Mechanical_Report T1.doc]

# Project

| First Saved | Thursday, April 19, 2018 |
| --- | --- |
| Last Saved | Wednesday, July 03, 2019 |
| Product Version | 14.5 Release |
| Save Project Before Solution | No |
| Save Project After Solution | No |

## Contents

- **Units**
- **Model (A4)**
  - Geometry
    - Part 1
  - Coordinate Systems
  - Mesh
    - Body Sizing
  - **Static Structural (A5)**
    - Analysis Settings
    - Loads
    - Solution (A6)
      - Solution Information
      - Results
        - Convergence
        - Convergence
        - Convergence
- **Material Data**
  - 爪子

## Units

TABLE 1

| Unit System | Metric (mm, t, N, s, mV, mA) Degrees rad/s Celsius |
| --- | --- |
| Angle | Degrees |
| Rotational Velocity | rad/s |
| Temperature | Celsius |

## Model (A4)

### Geometry

TABLE 2
Model (A4) > Geometry

| Object Name | *Geometry* |
| --- | --- |
| State | Fully Defined |
| **Definition** | |
| Source | E:\博士论文\英文论文\Structure, mechanics and material properties of claw cuticle from mole cricket 6-12\Plos One\静力学分析\收敛\T1_files\dp0\SYS\DM\SYS.agdb |
| Type | DesignModeler |
| Length Unit | Millimeters |
| Element Control | Program Controlled |
| Display Style | Body Color |
| **Bounding Box** | |
| Length X | 1.754 mm |
| Length Y | 2.503 mm |
| Length Z | 0.7836 mm |
| **Properties** | |
| Volume | 0.5979 mm³ |
| Mass | 7.354e-010 t |
| Scale Factor Value | 1. |
| **Statistics** | |
| Bodies | 1 |
| Active Bodies | 1 |
| Nodes | 67347 |
| Elements | 44362 |
| Mesh Metric | None |
| **Basic Geometry Options** | |
| Parameters | Yes |
| Parameter Key | DS |
| Attributes | No |
| Named Selections | No |
| Material Properties | No |
| **Advanced Geometry Options** | |
| Use Associativity | Yes |
| Coordinate Systems | No |
| Reader Mode Saves Updated File | No |
| Use Instances | Yes |
| Smart CAD Update | No |
| Attach File Via Temp File | Yes |
| Temporary Directory | C:\Users\316\AppData\Roaming\Ansys\v145 |
| Analysis Type | 3-D |
| Decompose Disjoint Geometry | Yes |
| Enclosure and Symmetry Processing | Yes |

TABLE 3
Model (A4) > Geometry > Parts

| Object Name | *Part 1* |
| --- | --- |
| State | Meshed |
| **Graphics Properties** | |
| Visible | Yes |
| Transparency | 1 |
| **Definition** | |
| Suppressed | No |
| Stiffness Behavior | Flexible |
| Coordinate System | Default Coordinate System |
| Reference Temperature | By Environment |
| **Material** | |
| Assignment | 爪子 |
| Nonlinear Effects | Yes |
| Thermal Strain Effects | Yes |
| **Bounding Box** | |
| Length X | 1.754 mm |
| Length Y | 2.503 mm |
| Length Z | 0.7836 mm |
| **Properties** | |
| Volume | 0.5979 mm³ |
| Mass | 7.354e-010 t |
| Centroid X | 1.678 mm |
| Centroid Y | 1.752 mm |
| Centroid Z | -0.5175 mm |
| Moment of Inertia Ip1 | 3.35e-010 t·mm² |
| Moment of Inertia Ip2 | 8.16e-011 t·mm² |
| Moment of Inertia Ip3 | 3.838e-010 t·mm² |
| **Statistics** | |
| Nodes | 67347 |
| Elements | 44362 |
| Mesh Metric | None |

### Coordinate Systems

TABLE 4
Model (A4) > Coordinate Systems > Coordinate System

| Object Name | *Global Coordinate System* |
| --- | --- |
| State | Fully Defined |
| **Definition** | |
| Type | Cartesian |
| Coordinate System ID | 0. |
| **Origin** | |
| Origin X | 0. mm |
| Origin Y | 0. mm |
| Origin Z | 0. mm |
| **Directional Vectors** | |
| X Axis Data | [ 1. 0. 0. ] |
| Y Axis Data | [ 0. 1. 0. ] |
| Z Axis Data | [ 0. 0. 1. ] |

### Mesh

TABLE 5
Model (A4) > Mesh

| Object Name | *Mesh* |
| --- | --- |
| State | Solved |
| **Defaults** | |
| Physics Preference | Mechanical |
| Relevance | 0 |
| **Sizing** | |
| Use Advanced Size Function | Off |
| Relevance Center | Coarse |
| Element Size | Default |
| Initial Size Seed | Active Assembly |
| Smoothing | Medium |
| Transition | Fast |
| Span Angle Center | Coarse |
| Minimum Edge Length | 0.9590 mm |
| **Inflation** | |
| Use Automatic Inflation | None |
| Inflation Option | Smooth Transition |
| Transition Ratio | 0.272 |
| Maximum Layers | 5 |
| Growth Rate | 1.2 |
| Inflation Algorithm | Pre |
| View Advanced Options | No |
| **Patch Conforming Options** | |
| Triangle Surface Mesher | Program Controlled |
| **Advanced** | |
| Shape Checking | Standard Mechanical |
| Element Midside Nodes | Program Controlled |
| Straight Sided Elements | No |
| Number of Retries | Default (4) |
| Extra Retries For Assembly | Yes |
| Rigid Body Behavior | Dimensionally Reduced |
| Mesh Morphing | Disabled |
| **Defeaturing** | |
| Pinch Tolerance | Please Define |
| Generate Pinch on Refresh | No |
| Automatic Mesh Based Defeaturing | On |
| Defeaturing Tolerance | Default |
| **Statistics** | |
| Nodes | 67347 |
| Elements | 44362 |
| Mesh Metric | None |

TABLE 6
Model (A4) > Mesh > Mesh Controls

| Object Name | *Body Sizing* |
| --- | --- |
| State | Fully Defined |
| **Scope** | |
| Scoping Method | Geometry Selection |
| Geometry | 1 Body |
| **Definition** | |
| Suppressed | No |
| Type | Element Size |
| Element Size | 5.e-002 mm |
| Behavior | Soft |

## Static Structural (A5)

TABLE 7
Model (A4) > Analysis

| Object Name | *Static Structural (A5)* |
| --- | --- |
| State | Solved |
| **Definition** | |
| Physics Type | Structural |
| Analysis Type | Static Structural |
| Solver Target | Mechanical APDL |
| **Options** | |
| Environment Temperature | 22. °C |
| Generate Input Only | No |

TABLE 8
Model (A4) > Static Structural (A5) > Analysis Settings

| Object Name | *Analysis Settings* |
| --- | --- |
| State | Fully Defined |
| **Step Controls** | |
| Number Of Steps | 1. |
| Current Step Number | 1. |
| Step End Time | 1. s |
| Auto Time Stepping | Program Controlled |
| **Solver Controls** | |
| Solver Type | Program Controlled |
| Weak Springs | Program Controlled |
| Large Deflection | Off |
| Inertia Relief | Off |
| **Restart Controls** | |
| Generate Restart Points | Program Controlled |
| Retain Files After Full Solve | No |
| **Nonlinear Controls** | |
| Force Convergence | Program Controlled |
| Moment Convergence | Program Controlled |
| Displacement Convergence | Program Controlled |
| Rotation Convergence | Program Controlled |
| Line Search | Program Controlled |
| Stabilization | Off |
| **Output Controls** | |
| Stress | Yes |
| Strain | Yes |
| Nodal Forces | No |
| Contact Miscellaneous | No |
| General Miscellaneous | No |
| Store Results At | All Time Points |
| Max Number of Result Sets | Program Controlled |
| **Analysis Data Management** | |
| Solver Files Directory | E:\博士论文\英文论文\Structure, mechanics and material properties of claw cuticle from mole cricket 6-12\Plos One\静力学分析\收敛\T1_files\dp0\SYS\MECH\ |
| Future Analysis | None |
| Scratch Solver Files Directory |  |
| Save MAPDL db | No |
| Delete Unneeded Files | Yes |
| Nonlinear Solution | No |
| Solver Units | Active System |
| Solver Unit System | nmm |

TABLE 9
Model (A4) > Static Structural (A5) > Loads

| Object Name | *Fixed Support* | *Force* |
| --- | --- | --- |
| State | Fully Defined | |
| **Scope** | | |
| Scoping Method | Geometry Selection | |
| Geometry | 1 Face | |
| **Definition** | | |
| Type | Fixed Support | Force |
| Suppressed | No | |
| Define By |  | Components |
| Coordinate System |  | Global Coordinate System |
| X Component |  | 0. N (ramped) |
| Y Component |  | 0.3376 N (ramped) |
| Z Component |  | -0.7692 N (ramped) |

FIGURE 1
Model (A4) > Static Structural (A5) > Force

### Solution (A6)

TABLE 10
Model (A4) > Static Structural (A5) > Solution

| Object Name | *Solution (A6)* |
| --- | --- |
| State | Solved |
| **Adaptive Mesh Refinement** | |
| Max Refinement Loops | 1. |
| Refinement Depth | 2. |
| **Information** | |
| Status | Done |

TABLE 11
Model (A4) > Static Structural (A5) > Solution (A6) > Solution Information

| Object Name | *Solution Information* |
| --- | --- |
| State | Solved |
| **Solution Information** | |
| Solution Output | Solver Output |
| Newton-Raphson Residuals | 0 |
| Update Interval | 2.5 s |
| Display Points | All |
| **FE Connection Visibility** | |
| Activate Visibility | Yes |
| Display | All FE Connectors |
| Draw Connections Attached To | All Nodes |
| Line Color | Connection Type |
| Visible on Results | No |
| Line Thickness | Single |
| Display Type | Lines |

TABLE 12
Model (A4) > Static Structural (A5) > Solution (A6) > Results

| Object Name | *Equivalent Elastic Strain* | *Equivalent Stress* | *Total Deformation* | *Equivalent Plastic Strain* |
| --- | --- | --- | --- | --- |
| State | Solved | | | |
| **Scope** | | | | |
| Scoping Method | Geometry Selection | | | |
| Geometry | All Bodies | | | |
| **Definition** | | | | |
| Type | Equivalent Elastic Strain | Equivalent (von-Mises) Stress | Total Deformation | Equivalent Plastic Strain |
| By | Time | | | |
| Display Time | Last | | | |
| Calculate Time History | Yes | | | |
| Identifier |  | | | |
| Suppressed | No | | | |
| **Integration Point Results** | | | | |
| Display Option | Averaged | |  | Averaged |
| **Results** | | | | |
| Minimum | 9.438e-006 mm/mm | 6.695e-002 MPa | 0. mm | 0. mm/mm |
| Maximum | 6.275e-003 mm/mm | 44.74 MPa | 2.523e-002 mm | 0. mm/mm |
| **Information** | | | | |
| Time | 1. s | | | |
| Load Step | 1 | | | |
| Substep | 1 | | | |
| Iteration Number | 1 | | | |

TABLE 13
Model (A4) > Static Structural (A5) > Solution (A6) > Equivalent Elastic Strain > Convergences

| Object Name | *Convergence* |
| --- | --- |
| State | Solved |
| **Definition** | |
| Type | Maximum |
| Allowable Change | 20. % |
| **Results** | |
| Last Change | 10.96 % |
| Converged | Yes |

FIGURE 2
Model (A4) > Static Structural (A5) > Solution (A6) > Equivalent Elastic Strain > Convergence

Model (A4) > Static Structural (A5) > Solution (A6) > Equivalent Elastic Strain > Convergence

|  | Equivalent Elastic Strain (mm/mm) | Change (%) | Nodes | Elements |
| --- | --- | --- | --- | --- |
| 1 | 5.623e-003 |  | 67347 | 44362 |
| 2 | 6.275e-003 | 10.96 | 110127 | 74567 |

TABLE 14
Model (A4) > Static Structural (A5) > Solution (A6) > Equivalent Stress > Convergences

| Object Name | *Convergence* |
| --- | --- |
| State | Solved |
| **Definition** | |
| Type | Maximum |
| Allowable Change | 20. % |
| **Results** | |
| Last Change | 9.472 % |
| Converged | Yes |

FIGURE 3
Model (A4) > Static Structural (A5) > Solution (A6) > Equivalent Stress > Convergence

Model (A4) > Static Structural (A5) > Solution (A6) > Equivalent Stress > Convergence

|  | Equivalent Stress (MPa) | Change (%) | Nodes | Elements |
| --- | --- | --- | --- | --- |
| 1 | 40.69 |  | 67347 | 44362 |
| 2 | 44.74 | 9.472 | 110127 | 74567 |

TABLE 15
Model (A4) > Static Structural (A5) > Solution (A6) > Total Deformation > Convergences

| Object Name | *Convergence* |
| --- | --- |
| State | Solved |
| **Definition** | |
| Type | Maximum |
| Allowable Change | 20. % |
| **Results** | |
| Last Change | 3.656e-002 % |
| Converged | Yes |

FIGURE 4
Model (A4) > Static Structural (A5) > Solution (A6) > Total Deformation > Convergence

Model (A4) > Static Structural (A5) > Solution (A6) > Total Deformation > Convergence

|  | Total Deformation (mm) | Change (%) | Nodes | Elements |
| --- | --- | --- | --- | --- |
| 1 | 2.522e-002 |  | 67347 | 44362 |
| 2 | 2.523e-002 | 3.656e-002 | 110127 | 74567 |

## Material Data

### 爪子

TABLE 16
爪子 > Constants

| Density | 1.23e-009 tonne mm^-3 |
| --- | --- |

TABLE 17
爪子 > Isotropic Elasticity

| Temperature C | Young's Modulus MPa | Poisson's Ratio | Bulk Modulus MPa | Shear Modulus MPa |
| --- | --- | --- | --- | --- |
|  | 7247 | 0.25 | 4831.3 | 2898.8 |
